# Transcranial near-infrared light in treatment of neurodegenerative diseases

**DOI:** 10.3389/fphar.2022.965788

**Published:** 2022-08-08

**Authors:** Damir Nizamutdinov, Chibueze Ezeudu, Erxi Wu, Jason H. Huang, S. Stephen Yi

**Affiliations:** ^1^ Baylor Scott and White Health, Neuroscience Institute, Neurosurgery, TX, United States; ^2^ Texas A&M University, College of Medicine, Neurosurgery, TX, United States; ^3^ Texas A&M University, School of Pharmacy, Pharmaceutical Sciences, TX, United States; ^4^ Department of Oncology, Dell Medical School, The University of Texas at Austin, TX, United States

**Keywords:** near infrared light (NIR), photobiomodulation (PBM), neurodegenerative diseases, transcranial light therapy, glymphatic, lymphatic

## Abstract

Light is a natural agent consisting of a range of visible and invisible electromagnetic spectrum travels in waves. Near-infrared (NIR) light refers to wavelengths from 800 to 2,500 nm. It is an invisible spectrum to naked eyes and can penetrate through soft and hard tissues into deep structures of the human body at specific wavelengths. NIR light may carry different energy levels depending on the intensity of emitted light and therapeutic spectrum (wavelength). Stimulation with NIR light can activate intracellular cascades of biochemical reactions with local short- and long-term positive effects. These properties of NIR light are employed in photobiomodulation (PBM) therapy, have been linked to treating several brain pathologies, and are attracting more scientific attention in biomedicine. Transcranial brain stimulations with NIR light PBM in recent animal and human studies revealed a positive impact of treatment on the progression and improvement of neurodegenerative processes, management of brain energy metabolism, and regulation of chronic brain inflammation associated with various conditions, including traumatic brain injury. This scientific overview incorporates the most recent cellular and functional findings in PBM with NIR light in treating neurodegenerative diseases, presents the discussion of the proposed mechanisms of action, and describes the benefits of this treatment in neuroprotection, cell preservation/detoxification, anti-inflammatory properties, and regulation of brain energy metabolism. This review will also discuss the novel aspects and pathophysiological role of the glymphatic and brain lymphatics system in treating neurodegenerative diseases with NIR light stimulations. Scientific evidence presented in this overview will support a combined effort in the scientific community to increase attention to the understudied NIR light area of research as a natural agent in the treatment of neurodegenerative diseases to promote more research and raise awareness of PBM in the treatment of brain disorders.

## 1 Introduction

Photobiomodulation (PBM), or low-power light therapy, applies red or near-infrared (NIR) light over the body’s surface for health benefits. Near-infrared emitting light (wavelengths between 800 nm and 2,500 nm). The emitted light can penetrate transcranially the physical barriers of the skin and skull and reach the brain parenchyma if an optical window is used between 650 nm and 1,200 nm without excessive heat generation. Biomedical reports demonstrate the ability of NIR light to stimulate biochemical processes that maintain a healthy brain state and can be beneficial in acute and chronic pathologic brain conditions (Enengl et al., 2020), (Sanderson et al., 2018), (Gomez et al., 2021), (Li et al., 2022). Recent reports shed more light on different biological and chemical features of NIR light and its potential in treating disease states, prompting further research in this area. Thus, one area of interest is the effect of NIR light on the mitochondrion as a significant target of light therapy in the cell (Karu, 2008; Zhang et al., 2015). Additional studies have shown cytochrome c oxidase, the transport complex IV, as the primary photoacceptor that absorbs infrared photonic stimulation (Wong-Riley et al., 2005; Rojas et al., 2008; Wang et al., 2017; Morse et al., 2021). This absorption of light energy leads to photoexcitation in the mitochondrion, which initiates a signaling cascade of cellular events that promote the production of adenosine 5′-triphosphate (ATP), modulates the cellular levels of signaling molecules like Ca^2+^, reactive oxygen species (ROS), and releases nitric oxide from cytochrome C oxidase (de Freitas and Hamblin, 2016; Pruitt et al., 2020). Tissue stimulation with NIR light has demonstrated secretion of vascular endothelial growth factor in the endothelial cells leading to angiogenesis and the formation of capillary-like structures (Tuby et al., 2006; Cury et al., 2013). Animal studies have also demonstrated that treatment with transcranial NIR (tNIR) light stimulates the brain’s glymphatic system flow, which promotes brain parenchymal homeostasis and clearance of excessive accumulation of metabolites along with protein aggregates from both deep and superficial brain regions in normal and different pathological conditions (Zinchenko et al., 2019; Zinchenko et al.,2020; Semyachkina-Glushkovskaya et al., 2021b; Xuan et al., 2022; Zhang et al., 2022).

Parkinson’s, Huntington’s, and Alzheimer’s are neurodegenerative diseases characterized by the accumulation of toxic proteins in various brain regions, leading to progressive loss of neurons over time due to associated pathophysiological changes. Thus, aggregations of Lewy bodies (containing alpha-synuclein) are observed in Parkinson’s disease, polyglutamine repeats and excess glutamine in the synaptic clefts in Huntington’s disease, and amyloid plaques are typical for pathologies associated with Alzheimer’s disease (Scheltens et al., 2016; Cieri et al., 2017; Jimenez-Sanchez et al., 2017). These protein aggregates accumulate to cytotoxic levels and directly possess the neurotoxic effect on neurons or can disrupt mitochondrial proteins like cytochrome c oxidase, ATP synthase, membrane translocases, and voltage-dependent anion channels (Dong et al., 2009; Hernandez-Zimbron et al., 2012; Pinho et al., 2014; Magri and Messina, 2017). Impaired functions of these proteins lead to decreased mitochondrial membrane potential, low ATP production, and increased oxidative stress and trigger apoptosis of neurons (Zhang et al., 2010; Liang et al., 2012; Chen and Zhong, 2014). Protein aggregates can also activate microglial cells in the brain, leading to the chronic release of pro-inflammatory cytokines that exert toxic effects on neurons (Pagani and Eckert, 2011; Koper et al., 2018; Marogianni et al., 2020; Victoria et al., 2020). Many believe that mitochondrion dysfunction may be a central contributing factor to neurodegenerative disease development. Therefore, scientists explore and evaluate the response to light therapy.

Because of promising outcomes and the low probability of developing complications, the tNIR light research area has recently attracted attention in the scientific community. New animal studies and clinical trials assess the effects of tNIR light stimulation in various diseases. Some studies report that treatment with tNIR light reduces hyperphosphorylated tau, neurofibrillary tangles, and amyloid-beta plaques in transgenic animals (Purushothuman et al., 2014; Zinchenko et al., 2020; Yang et al., 2022). Studies in drug-induced PD animal models demonstrated safety and significant improvement in fine motor skills and mobility (Salehpour and Hamblin, 2020; Liebert et al., 2021). Some studies also show that tNIR light treatment mitigates the loss of dopaminergic fibers and protects neurons against alpha-synuclein-induced toxicity (Oueslati et al., 2015; El Massri et al., 2017).

tNIR light-emitting technologies also improved in recent years. They made it possible for investigators not only to change the positioning of light-emitting modules on a patient’s head to enhance the treatment and likelihood of reaching the target area of the brain depending on disease state and location of pathology but also to make it more versatile in controlling and adjusting power, frequency of emitting light, the intensity of a beam, or change of pulse rate. This can create a unique and personalized therapeutic unit that can be adapted for use in different clinical conditions or disease states depending on the need or areas of interest for light penetration depth and emission cycles during treatment sessions. In addition, some devices were designed to be used by study subjects at the convenience of their home without direct supervision by research personnel (Nizamutdinov et al., 2021). Some studies report that new tNIR light-emitting devices have improved safety, convenience to use, and easy operation of the device by study subjects or caregivers, which offers more versatility and flexibility in future studies in this area of research (Nizamutdinov et al., 2021).

The use of NIR light treatment by patients in clinical or home settings comes down to two major sets of variables: 1) the light-emitting device-related, which is tuned by research or clinical personnel to target a particular area of the brain depending on the disease; and 2) specific therapeutic protocol related, which also depends on the type of pathology and stage of the disease. These variables can add endless combinations of parameters to the field where the same disease can be approached with various wavelengths of light, adjusted power of emission, or pulse rate for delivery to a target tissue, and of course, can add variables with treatment protocol like duration of each session, a number of applications, duration of therapy and so on creating confusion in the field and total disarray. That is where dosimetry can be very helpful in standardizing different approaches to common ground and influencing the order of things to get to the goal of the treatment outcome (Pitzschke et al., 2015). The purpose of dosimetry is to help deliver a particular amount of light stimulation per area of the target tissue to trigger the same signaling cascade or cell receptors activation to initiate a beneficial cascade of effects in the target tissue despite the difference in physical characteristics of a PBM light. The unique part of dosimetry is that a similar quantitative outcome can be achieved using different devices and light sources (800 nm, 1,000 nm, or 1,200 nm) concerning the ability of light to reach a certain depth. The adjustments can be made to light stimulation duration time or light emission power which can be reflected in similar outcomes across the board. This is where standardization of treatment across the entire field can become a reality for investigators with different approaches to achieve the same result on a known scale of quantitative light measurement for the light wavelength (Pitzschke et al., 2015; Khan and Arany, 2016).

This review will provide a scientific overview of the current findings using tNIR light stimulations, mechanism of action, and benefits of tNIR light stimuli in treating neurodegenerative diseases. We discuss proposed cellular mechanisms of action and describe the benefits of this treatment in neuroprotection, cell preservation/detoxification, anti-inflammatory properties, and regulation of brain energy metabolism. This review will explain the effects of tNIR light on the brain glymphatic and lymphatics systems and how these effects can be adopted in treating chronic neurodegenerative and other neurological diseases as potentially promising therapeutic options. It will also discuss the current challenges and limitations of some techniques or therapeutic approaches and future directions in the field of PBM.

## 2 Mechanism of action of transcranial near-infrared light brain stimulation

The tNIR light stimulations of brain parenchyma affect several areas and trigger cascades of key signaling regulatory molecules that have immediate local and distant systemic responses.

Local brain responses to tNIR light stimulations include 1) elevating intracellular ATP production and stimulating mitochondrial function; 2) management of local oxidative stress; 3) local vasodilation (both vascular and lymphatic); 4) creation of new and restoration of damaged synapses; 5) stimulation of new neuronal growth; 6) neuroprotective and cell-protective properties; 7) stimulation of new vascular growth.

Systemic responses to tNIR light stimulation include 1) stimulation of anti-inflammatory and immunological responses.

Intracellular ATP production is the most commonly studied and supported effect of PBM. It was reported that PBM stimulates a significant increase in intracellular levels of nucleotide triphosphates. This coincides with increased mitochondrial membrane potential and mitochondrial function (Karu, 2010; de Freitas and Hamblin, 2016).

Oxidative stress management occurs through counteracting ROS observed in excessive amounts during neurodegenerative diseases and other neurological conditions (Maurya et al., 2016; Tramutola et al., 2017). One known mechanism is regulating cytokine-inducible nitric oxide synthase (iNOS). When iNOS is controlled by antioxidant secretion stimulated by PBM, it leads to decreased levels of nitric oxide, which results in a decrease in reactive nitrogen species and oxidative stress.

Local vasodilation is also a widely supported effect of PBM in the scientific community (Mungrue et al., 2002). Animal studies demonstrate profound secretion of nitric oxide in response to PBM stimulation. The presence of NO in local vascular and lymphatic circulation as a result of this stimulation leads to vasodilation, promotes free, unobstructed flow of blood and lymphatic fluid, gas exchange, and overall brain circulation through vascular and lymphatic systems (Ahmed et al., 2011; Semyachkina-Glushkovskaya et al., 2021a). Improved flow-through brain lymphatics also support immune function in the brain, improve immune cell exchange rate in chronic inflammatory conditions and promote better anti-inflammatory properties of innate immunity (Salehpour et al., 2022).

Synaptogenesis and restoration of damaged synapses in chronic neurodegenerative disease or acute or chronic traumatic brain injury occur after PBM stimulation through regulation of brain-derived neurotrophic factor (BDNF) (Meng et al., 2013). BDNF helps maintain synapses by promoting their growth and acceleration of synaptic contacts, which can be achieved by downregulation of synapsin-1 protein (Marte et al., 2017). Another mechanism by which tNIR light stimulation promotes new synapse formation is by triggering stem cells (Xuan et al., 2014).

Stem cell stimulation by PBM treatment is also linked to studies that observed the formation of neurons (neurogenesis) (Xuan et al., 2014). An animal study reported improvement of neurogenesis in the damaged brain parenchyma after stimulation with PBM. The BDNF mentioned above also contributes to neuronal growth. Activation of that signaling cascade goes through the intracellular ERK/CREB signaling pathway stimulated by PBM (Meng et al., 2013).

Stimulations with NIR light showed neuroprotective for brain parenchyma and surrounding tissues. This function is achieved by activating the protein kinase B (AKT) signaling pathway. AKT signaling in different tissue types is proven cell-protective (Liang et al., 2012). However, to be activated, it should go through glycogen synthase kinase 3β (GSK3 β) mediator, which is responsible for switching between apoptotic (Bax signaling) or cell-protective/neuroprotective (AKT cascades) (Zhang et al., 2010). Another mechanism of neuroprotection can be achieved by PBM activating the extracellular signal-related kinase (ERK) signaling cascade. Once activated, ERK translocates to the nucleus with nuclear accumulation of FOXM1 molecule, inhibiting p21 protein expression and slowing cellular senescence in neurons (Ling et al., 2014). This is the indirect neuroprotective effect on the brain by PBM stimulation.

Stimulating new blood vessel formation by tNIR light stimulations is achieved by increasing vascular endothelial growth factor expression or decreasing matrix metalloproteinase 2 activity (Cury et al., 2013).

Anti-inflammatory effects are mainly achieved by PBM inhibition of cyclooxygenase2 enzyme (COX-2) and by inhibiting transcription factor NF-kB pathway. Both are well-reported pro-inflammatory enzyme and pro-inflammatory gene expression cascades, respectively (Lim et al., 2013). In addition, PBM stimulation also helps manage innate immune response by regulating other pro-inflammatory cytokines and improves lymphatic fluids flow in brain lymphatic and glymphatic drainage systems (Semyachkina-Glushkovskaya et al., 2020a; Salehpour et al., 2022).

## 3 Effects of transcranial near-infrared light stimulations in chronic traumatic brain injury syndrome

Traumatic brain injuries (TBI) happen when direct or transmitted external forces cause damage to the brain, often observed in falls, sports injuries, road accidents, and assaults. TBIs are classified as mild, moderate, or severe depending on the clinical spectrum and assessment of patient factors, including with/without loss of consciousness, altered mental state, and post-trauma amnesia (Blennow et al., 2016). TBI is more common in the younger population than atherosclerotic events like stroke and considerably impacts the healthcare industry. Studies estimate that nearly 2 million head injuries occur in the US annually, resulting in 283,000 hospitalizations and 53,000 deaths (Sosin et al., 1996). The impact of trauma disrupts physiologic pathways leading to inflammation, oxidative stress, mitochondrial dysfunction, and increased vascular permeability resulting in brain neuronal death and the spread of necrosis over brain parenchyma (Zink et al., 2010). Unfortunately, there is no universally accepted treatment for TBI, and novel therapeutics like PBM reported to increase tissue oxygenation, reduce neuroinflammation, and induce neurogenesis is a promising intervention to treat traumatic brain injuries.

Many research investigations have reported promising results for treating acute TBI using PBM in animal models. For example, one group of researchers induced a closed-head injury in mice with a weight-drop device. It delivered 2 minutes of irradiation using an 808 nm laser 4 hours after the TBI induction. There was a significant improvement in neurofunction and minor loss of cortical tissue in the PBM group compared with the control group (Oron et al., 2007). A different team of researchers investigated the effects of varying wavelengths of laser light (665, 730, 810, 980 nm) on the scalp 4 hours post-TBI. Results showed significant improvement in the 665 and 810 nm laser groups compared to control. However, there were no significant improvements in the 730 and 980 nm groups, attributed to cytochrome c oxidase having absorption bands in the 665 and 810 nm regions (Wu et al., 2012). Another team of researchers conducted similar studies using a controlled cortical impact device to induce TBI in mouse models. After treatment with PBM, immunofluorescence of brain sections revealed increased neuroprogenitor cells, increased brain-derived neurotrophic factor (BDNF), and improved learning and memory (Xuan et al., 2015).

Unlike animal studies in acute TBI, most of the human case studies of PBM in TBI are assessed in chronic settings. For example, one open protocol study used light therapy in eleven chronic mild TBI participants (26–62 years of age), ranging from 10 months to 8 years post mild TBI injury. This study revealed significant improvements in the Stroop test for executive function and California Verbal Learning Test (CVLT)-II. Participants from this study also reported increased sleep, reduced post-traumatic stress disorder (PTSD) symptoms if present at baseline, and improved social and occupational functions (Naeser et al., 2014). Another report on patients with moderate TBI and cognitive dysfunction treated with 18 sessions of tNIR therapy demonstrated improvement in measures of depression, verbal memory, executive function, and sleep efficiency (Bogdanova et al., 2014).

Another research team studied the effects of PBM in patients with an average span of 9.3 years after the injury. Participants received ten treatments over 2 months using NIR laser with 810 and 910 nm and reported improved cognition, mood dysregulation, anxiety, irritability, headache, and sleep disturbance (Morries et al., 2015). A similar study with ten adult patients classified with severe TBI who received PBM therapy three times a week for 6 weeks revealed increased cerebral blood flow and improved hemodynamic response (Carneiro et al., 2019). Additional similar published studies further provide evidence of the beneficial effect of PBM therapy in chronic TBI (Nawashiro et al., 2012; Hipskind et al., 2018; Chao et al., 2020).

## 4 Effects of transcranial near-infrared light stimulation on lymphatic and glymphatic systems and contribution to prevention or treatment of neurodegenerative diseases

The unknown mechanism of interstitial fluid clearance from the extracellular brain space of the CNS led researchers to identify the glymphatic pathway in animal models (Iliff et al., 2012). Before discovering the glymphatic system, interstitial fluid transport was previously attributed to diffusion; however, recent animal research indicates the presence of the lymphatic drainage system from the brain parenchyma into cervical lymph nodes (Casley-Smith et al., 1976). The glymphatic system, which is most activated during sleep, is responsible for other physiological functions such as promoting glial signaling, regulating brain response to neuroinflammation, and stimulating the transport of apoprotein E, which plays a role in synaptic plasticity (Rangroo Thrane et al., 2013; Xie et al., 2013; Achariyar et al., 2016; Chen et al., 2021). Impaired glymphatic pathway clearance is diminished in diseases like Alzheimer’s and Parkinson’s, explained by the common feature of accumulation of the protein aggregates in patients (Rasmussen et al., 2018; Zou et al., 2019). Pathological incidents like traumatic brain injury and stroke also affect clearance from the glymphatic system (Ren et al., 2013; Rasmussen et al., 2018). A plausible understanding from research studies indicates that neuroinflammation leads to reactive astrogliosis, which results in the loss of aquaporin channels AQP4 in astrocytes correlating to a decrease in glymphatic flow (Kress et al., 2014; Jha et al., 2018). There is also a physical blockade of perivascular flow by infiltrating immune cells during inflammation, leading to the accumulation of cytokines, metabolic products, and inflammatory mediators, perpetuating neuroinflammation (Dickson and Rogers, 1992; Zhou et al., 2009).

A series of animal model experiments have identified transcranial PBM’s effect on glymphatic drainage in animal studies. One group of researchers investigated the effects of PBM on lymphatic vessel contractility and pumping, which characterize the mechanism of waste clearances and fluid transport from the brain, resulting in a decrease in local edema. tNIR stimulation increases the diameter of meningeal lymphatic vessels by inducing vasodilation and the relaxation of vessels (Semyachkina-Glushkovskaya et al., 2020b). Further, investigators studied the effects of tNIR on the drainage function of meningeal lymphatic vessels (MLVs) by injecting gold nanorods (GNRs) into different brain regions. Results show increased GNRs transport from the cortex by 55.7-fold, cisterna magna, hippocampus, and lateral ventricles by −14.78- fold, 4.8-fold, and 2.3-fold, respectively (Semyachkina-Glushkovskaya et al., 2020a). Other related studies also showed that transcranial use of quantum-dot laser at 1,267–1,268 nm PBM mediated the opening of the blood-brain-barrier (BBB) and promoted the transport of macrophages from lymphatic vessels into surrounding tissues by increasing lymphatic permeability (Semyachkina-Glushkovskaya et al., 2020b). Activation of lymphatics by PBM also increased drainage and stimulated the proliferation of glioma cells (Semyachkina-Glushkovskaya et al., 2021a). This group claims no heating effect generated by laser at 1,300 nm light which is less scattering and could penetrate deeper into the brain tissue compared to light ranging from 800 to 1,100 nm (Wang et al., 2018; Semyachkina-Glushkovskaya et al., 2021a). One proposed mechanism by the group is the activation of NO synthesis in lymphatic cells, which causes vasodilation of lymphatic vessels and leads to increased permeability and, as a result, drainage function of brain lymphatics. At the same time, light stimulates the contractility of lymphatic vessels and promotes the pumping function of lymphatic vessels, which carries lymph away and large molecules from the brain (Semyachkina-Glushkovskaya et al., 2021a).

Additional studies on the lymphatic pathway of red blood cells (RBC) after intraventricular hemorrhage showed that mice treated with PBM had increased clearance of RBC from the ventricles into deep cervical lymph nodes via transport through MLVs which led to a quicker recovery of intracranial pressure and a decrease in mortality by 1.57-fold (Li et al., 2020). Also, studies on AD models demonstrate that transcranial PBM increases energy metabolism in the brain, which activates the lymphatic system and significantly increases the clearance of amyloid-beta plaques from the brain via meningeal lymphatic vessels (Zinchenko et al., 2019; Semyachkina-Glushkovskaya et al., 2021b). Thus, in Table 1 authors demonstrated the effects of 1,267 nm light with variable fluence on the brain surface. The impacts of 18J/cm^2^ and 25J/cm^2^ were ineffective; however, 32J/cm^2^ and 39J/cm^2^ were effective against intracranial amyloid-beta plaques accumulation. The light at 39J/cm^2^ reported negative morphological changes in brain vessels with brain edema formation and scalp temperature at 37°C. The 32J/cm^2^ light effects were optimal for amyloid-beta plaques management with scalp temperature at 33°C (Zinchenko et al., 2019).

## 5 Effects of transcranial near-infrared light stimulations in Alzheimer’s disease and Alzheimer’s disease-related dementia

Alzheimer’s disease (AD) is a neurodegenerative disease associated with progressive memory impairment, cognitive deficits, and behavioral degradation. The condition commonly targets the cerebral cortex, parietal and temporal lobes with the development of atrophy and metabolic dysfunction associated with pathologic buildup of beta-amyloid peptide (β42), hyperphosphorylated tau protein, neurofibrillary tangles, and formation of toxic amyloid plaques (Hennessy and Hamblin, 2017).

The scientific community responded to the pressing need to develop a new therapeutic remedy for AD and Alzheimer’s disease-related dementia (ADRD). This effort resulted in several animal and clinical studies in recent years focused on the effects and mechanisms of PBM treatment of AD pathology. Some animal studies in the transgenic mouse AD model demonstrated strong beneficial tNIR treatment effects. Thus, AD transgenic animals were treated with tNIR, and pathological accumulation of toxic plaques and other markers of brain cell damage was quantitatively evaluated (Purushothuman et al., 2014; Purushothuman et al., 2015). Results of the study demonstrated a significant decrease in the size and number of amyloid-β plaques in different brain regions of AD animals (Purushothuman et al., 2014; Purushothuman et al., 2015). Another study using the AD pathological mice model reported a change in signaling cascade molecules in brain cells in response to PBM treatment. The study revealed potential therapeutic value by achieving regulation of the JNK3 kinase pathway and stabilizing the MKP7 cascade, which is believed to be brain-specific isoforms associated with neurodegeneration cascades. Observed molecular changes translated into the rescue of memory loss deficit, amyloid load reduction, synaptic loss, and neuroinflammation in AD model transgenic APP/PS1 mice treated with PBM (Shen et al., 2021). The beneficial findings observed in AD model animal studies draw more attention and build an evidence-based foundation in the biomedical community to make more clinical trials. Thus, clinical trials using tNIR have reported improvement in cognitive function and memory, which suggests a decrease in the speed of progression of disease and treatment contributes to slow neurodegeneration (Lim, 2013). Our team concluded a randomized, double-blind, and placebo-controlled clinical trial for treating ADRD with a helmet device emitting tNIR light at 1,060–1,080 nm wavelength set at low intensity twice daily for 8 weeks. The helmet device used transcranial and trans-orbital routes of NIR light delivery. Results revealed improvements in MMSE test, clock drawing tests, cognition, level of concentration on tasks, memory, and decreased level of anxiety in ADRD diagnosed patients (Nizamutdinov et al., 2021).

## 6 Effects of transcranial near-infrared light stimulations in Parkinson’s disease

Parkinson’s disease (PD) is neurodegenerative pathology with a gradual decline of dopaminergic neurons in substantia nigra. Pathology progressively affects the ability to make movements (Moro et al., 2013). There is a lot of support in the scientific community for the mitochondrial theory of PD (mitochondrial dysfunction linked to PD). Some genetic mutations associated with *PINK1* and *PRKN* genes have been linked to early-onset forms of PD (Kitada et al., 1998; Valente et al., 2004). The combination of mitochondrial dysfunction and insufficiency with age-related genomic instability creates more failures of neuronal cells on the level of bio-energetic demand. The most vulnerable cells with genetic deficiencies continue to contribute to premature loss of excessive numbers of neurons and cause the most prominent clinical presentation (Sterky et al., 2011). Therefore the strategy to improve mitochondrial function in a vulnerable group of neurons is detrimental and received much support in the scientific community for its potential to be a therapeutic solution. On this basis, several animal studies were launched with a drug-induced PD model using PBM treatment. Studies revealed positive changes associated with decreased expression of hyperphosphorylated tau in animals, improved autonomous activities, and, therefore, beneficially affected the course of disease (Moro et al., 2013; Purushothuman et al., 2013; Reinhart et al., 2015). A study with Macaque Monkeys with drug-induced PD treated with NIR resulted in positive outcomes with improved posture, activity, and facial expressions in animals compared to the untreated group (Moro et al., 2016).

A clinical trial using tNIR in patients diagnosed with PD reported positive results with improved cognition and motor function after 2 weeks of treatment (Maloney et al., 2010). Another proof-of-concept clinical study using a combination of transcranial and remote treatment with PBM for at least 12 weeks reported statistically significant improvement in mobility, cognition, fine motor skills, and dynamic balance (Liebert et al., 2021). Another clinical trial using combined transcranial and intra-oral therapy with NIR light stimulations demonstrated no significant changes in performed measures but concluded to have at least 4 weeks duration of treatment and 2-3 therapeutic sessions per week before any improvements in outcomes could be evident (Bullock-Saxton et al., 2021).

## 7 Potential challenges and future directions

Field of PBM and use of NIR light for treating various neurological conditions demonstrate many beneficial effects even in chronic, established disease states. Therefore, they have many promises with little to no reported side effects. Thus, the NIR light area of research is rapidly evolving and is a very appealing and forgiving area for experimentation. The biomedical field achieved noticeable progress in the evolution of NIR light use in recent years, but this does not come without challenges.

The most commonly discussed limitation of treating PD with tPBM is the anatomical location of the primary target area (substantia nigra pars compacta). Because of location, which is relatively deep inside of brain tissue and potentially may not be reachable by some light-delivering systems or approaches of PBM. More complicated light-delivering methods through wirelessly powered device implantation into the third ventricle and the sphenoid sinus locations currently present challenges on technical and biological levels (Foo et al., 2020).

The main challenge with this technology we face is the lack of regulation, educational information, and general public awareness in the field. We must admit that technology has great potential and offers flexibility and forgiveness to use at the convenience of the home without supervision. However, the general public should treat it as a therapeutic intervention even though it uses a non-invasive route. With promising scientific publications in the field, it is still not a thoroughly investigated area of research with many variables which include and are not limited to wavelength of the light, power, intensity of the light, frequency of pulse (if any), location and position of light-emitting modules in respect to the brain, routes of administration (transcranial, trans-orbital, trans-nasal, or pharyngeal) duration of each treatment session, number of sessions per day, the longevity of the course, preexisting conditions, age, the color of the skin, the color of the hair to name a few. Because of many variables associated with technology and patient, developing a working therapeutic protocol across PBM or NIR light discipline creates a challenge. Another challenge associated with it is the general publics’ take-home message based on the published information.

Regarding this, several vendors in the current marketplace are selling NIR light-emitting devices to the public (no prescription is needed). However, because of the variables mentioned above, the absence of FDA approval, and the lack of established therapeutic protocol general public should be cautious of the harm these light-emitting devices can potentially cause when misused or overused. Thus, a possible challenge to the field presents potential public misconception and mistrust of promising technology based on the experience of using not approved devices or inappropriate therapeutic protocols.

Future directions of this field should focus on developing approaches to overcome existing challenges and limitations without compromising the safety of our patients. More research using tNIR light should be done to treat brain oncology and cancer treatment-induced side effects management. tNIR light stimulations can be used to improve the permeability of BBB for better drugs delivery to the target area of brain cancer, can help manage local inflammatory response, and activation of anti-apoptotic and cell-protective signaling cascades (Zhang et al., 2010; Liang et al., 2012; Bensadoun, 2018; Semyachkina-Glushkovskaya et al., 2021a). More research focusing on dosimetry in the PBM field can help secure the reproducibility of treatment protocols even with the use of different PBM devices and therapeutic modalities. The biomedical field needs more animal studies and large placebo-controlled randomized clinical trials with more statistically significant data to draw a scientific conclusion and test the limits of promising tNIR light treatment technology in medicine.

## 8 Summary

Recent studies focused on NIR light effects on brain physiology and pathophysiology made it easy to conclude that PBM with NIR light stimulation has much potential as a future option for treating acute and chronic brain pathologies, including neurodegenerative disorders. The use of NIR light in different settings, power, intensity, wavelength, and routes of light delivery to the target tissue to achieve various study objectives represents the versatility of this technology. Many reports claim safety, the convenience of use, and a wide therapeutic window to use in multiple applications. Nevertheless, of course, it is an emerging technology, and it is not without limitations. High-quality, large placebo-controlled randomized clinical trials are warranted to understand better the role of tNIR light in the management of neurodegenerative diseases.
